# Evaluation of HER2/neu Expression in High-Grade Endometrial Carcinoma and Its Clinicopathological Correlation

**DOI:** 10.30699/ijp.2019.90831.1867

**Published:** 2019-09-22

**Authors:** Soheila Sarmadi, Narges Izadi-mood, Nazanin Mansourzadeh, Dorna Motevalli

**Affiliations:** 1 *Department of Pathology,Yas Hospital, Tehran University of Medical Sciences,Tehran, Iran*; 2 *Department of Pathology, Imam Khomeini Hospital, Tehran University of Medical Sciences, Tehran, Iran*; 3 *Sina Hospital, Tehran University of Medical Sciences, Tehran, Iran*

**Keywords:** Human epidermal growth factor receptor 2, Endometrial carcinoma, Prognosis, HER2/neu expression

## Abstract

**Background & Objective:**

Endometrial carcinoma (EC) has been traditionally classified into two distinct categories of low-grade and high-grade. Type I (low grade) EC, which constitutes the majority of cases, is linked to estrogen-related molecular pathways. But type II (high-grade) EC accounts for 10-20% of cases and behaves in an aggressive way. Pathologic and biological features of type II EC have not been fully elucidated yet. Several investigations have demonstrated HER2/neu expression and amplification in type II EC, especially papillary serous carcinoma (PSC). This study assessed HER2/neu expression in high-grade EC as well as its association with other clinical and histopathological prognostic factors.

**Methods:**

In this cross-sectional study, we performed HER2/neu immunohistochemical (IHC) staining in 37 high-grade EC cases with histological diagnostic categories of PSC (n=23), clear cell carcinoma (CCC) (n=9), and carcinosarcoma with high-grade carcinomatous component (PSC, CCC, grade 3 endometrioid carcinoma, or unclassified high-grade adenocarcinoma) (n=5). All patients were followed for 2-9 years in order to evaluate their disease-free survival (DFS) and overall survival (OS) during study period (2005-2014).

**Results:**

HER2/neu IHC staining was positive in 12 patients (32.4%) including 8/23 (34.8%) PSC, 2/9 (22.2%) CCC, and 2/5 (40%) carcinosarcoma cases. There was no statistically significant difference between HER2/neu expression and DFS or OS of the patients (*P*>0.05).

**Conclusion:**

We observed that HER2/neu is expressed in one-third of high-grade ECs. This ancillary test is supportive in follow-up of patients with high-grade ECs.

## Introduction

Endometrial carcinoma (EC) accounts for the majority of gynecological malignancy cases. It has been traditionally divided into two subgroups of types I and II, based on their different pathogenic pathways and prognosis. Type I EC occurs in association with hyperestrogenic state in which tumors are commonly well differentiated. This type of EC mainly occurs in background of endometrial hyperplasia and it usually responds to hormone therapy, whereas type II EC is not related to hyperestrogenic state and usually occurs in older age groups in the setting of atrophic endometrium or sometimes endometrial polyp, and typically presents as high-grade tumors with poor prognosis (1-6). High-grade ECs are composed of grade 3 endometrioid endometrial carcinoma (EEC3) and mainly non-endometrioid endometrial carcinomas including papillary serous carcinoma (PSC), clear cell carcinoma (CCC), undifferentiated carcinomas, and malignant mixed mullerian tumors (MMMT) (7). They have a strong propensity to lymph node metastasis, as well as spreading and implanting into areas such as adnexa and peritoneum. While CCC commonly metastasizes into lymph nodes, EEC3 metastasizes first to pelvic organs and para-aortic lymph nodes (5). High-grade EC comprises only about 25–30% of all EC cases, but accounts for 70–75% of EC-related mortality (7). Due to differed biological characteristics, very aggressive behavior, high recurrence rate, poor prognosis, as well as difficulty in histopathological diagnosis of such tumors, the immunohistochemical (IHC) assessment may be useful (3, 4, 8, 9).

The mentioned two types of EC are characterized by distinct genetic instability and different molecular genetic pathways. Amplification and overexpression of HER2/neu have been shown to have a significant role in the pathogenesis of cancers in various organs, including carcinomas of breast, ovary, stomach, and esophagus (10). The reported rates of HER2/neu overexpression in PSC range between 14-80% and this range also varies between 21-47% in other studies using HER2/neu amplification methods such as fluorescence in situ hybridization (FISH) (10). Other studies included only few cases of CCC and overexpression of HER2/neu was seen in 33-66% and 22-38% using IHC and amplification detection methods, respectively (10). Amplification of this gene is accompanied with high-grade, rapid progression, increased invasion, advanced disease, poor prognosis, and partial resistance to endocrinological and chemotherapy treatments (11). Several recent studies have been focused on the pathogenetic mechanism and prognostic value of HER2/neu expression in EC, especially in PSC, which provide basis for targeted immunotherapy in selected cases (12, 13). 

This study aimed to assess the overexpression of HER2/neu by IHC method in patients with high-grade EC and evaluate its correlation with the clinicopathological features to provide practical therapeutic and prognostic clues for pathologists and clinicians.

## Materials and Methods

In this cross-sectional study, we enrolled 37 patients with non-endometrioid high-grade carcinoma who underwent hysterectomy and staging according to the FIGO system (surgical staging) in two teaching hospitals, YAS and Imam Khomeini, affiliated to Tehran University of Medical Sciences, Tehran, Iran in the period of 2005-2014. Before conducting the data collection phase, the study was reviewed and approved by the Ethics Committee of the University. Hematoxylin and eosin stained slides and selected paraffin embedded tissues were obtained from the archival pathology files. Histopathologic data including the histologic type of tumor, tumor size, depth of myometrial invasion, lymph-vascular and perineural invasion, parametrial involvement, presence of endometrial polyp, and FIGO stage were reviewed. Final diagnosis was done according to the histomorphological criteria.

 Data regarding overall survival (OS) rate of 3-5 years and DFS were collected from the patients' clinical files and follow-up data. Patients with high-grade EC were included in this study but patients with other EC types and those with incomplete follow-up were excluded.

**Immunohistochemistry**

Paraffin embedded tissue sections with 5 μm thickness were prepared for IHC of HER2/neu biomarker using the HercepTest (Dako, Glostrup, Denmark) and according to the manufacturer's protocols.

**Scoring of the Results**

**Fig. 1:**
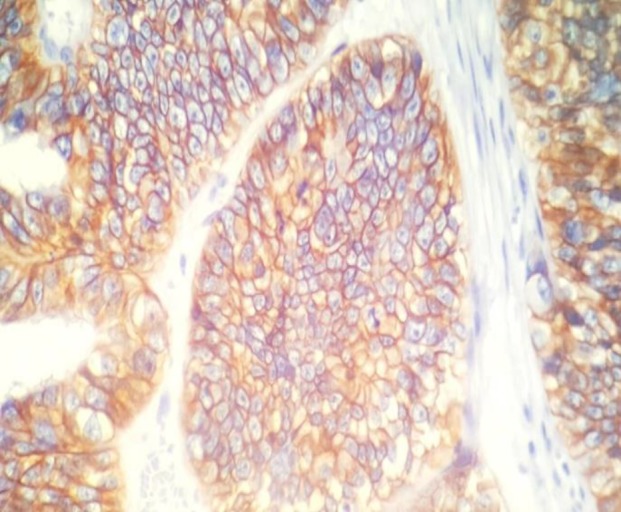
HER2/neu immunostaining score 3+

The results of IHC staining were evaluated according to the percentage of positive neoplastic cells. Semi-quantitative scoring of HER2/neu was done as follows: Score 0: no immunostaining/membrane staining in less than 10% of neoplastic cells; Score 1+: weak staining in more than 10% of neoplastic cells in only portions of the membrane; Score 2+: weak/ moderate circumferential membranous staining in >10% of tumor cells; Score 3+: strong complete membranous staining in more than 10% of tumor cells. Meanwhile, scores equal to 2+ and 3+ were considered as HER2/neu positive (Figure 1). HER2 expression was scored on epithelial component in carcinosarcoma cases.

**Fig. 2:**
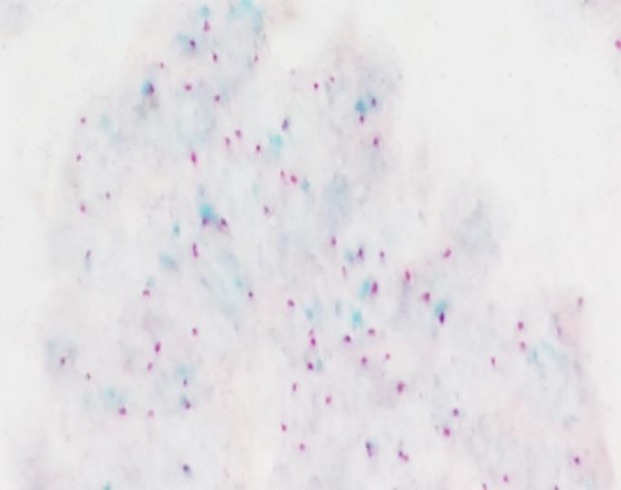
CISH study

All cases with 1+ and 2+ scores were confirmed with chromogenic in situ hybridization (CISH method). Positive HER2/neu amplification using CISH was defined as dual probe HER2/chr17 ratio equal or more than 2.0 with any average HER2 copy number. In tumors with marginal HER2/chr7 ratio of 1.8 to 2.2, another area was selected and additional 20 nuclei were scored and the average results were calculated (Figure 2).

**Statistical Analysis**

Descriptive statistics like Spearman rank correlation analysis were performed using SPSS 21 (SPSS Inc., Chicago, Illinois, USA) and P*-*value less than 0.05 was considered significant. Kaplan-Meier survival curves for progression-free survival (PFS), OS, and disease specific survival (DSS) were evaluated using a log-rank test. Also Chi-Square, Fisher, and independent-samples t-tests were used.

## Results

We identified a total number of 37 patients with high-grade EC. This included 62.2% (23) PSC, 24.3% (9) CCC, and 13.5% (5) carcinosarcoma cases with high-grade carcinomatous components (PSC, CCC, grade-3 endometrioid carcinoma, or unclassified adenocarcinoma). The mean age ± SD was 59.4±10.2 years. The mean diameter of tumor was 3.7±2.4 cm. The topographic characteristics of the cases are summarized in Table 1.

No significant correlation between the stage as well as type of tumor and HER2/neu overexpression was identified (*P*>0.05) (Table 2). All three cases with omental involvement and 5 cases with pelvic lymph node involvement were of PSC type and they showed HER2/neu negativity. Five cases with adnexal involvement were PSC histologic type; and while one of them showed HER2/neu overexpression, 4 others were HER2/neu negative. Cervical stromal involvements were identified in 11 patients including 2 cases of carcinosarcoma (HER2/neu overexpression in all), one CCC (HER2/neu negative), and 8 PSC cases (3 cases with HER2/neu overexpression and 5 HER2/neu negative).

**Fig. 3:**
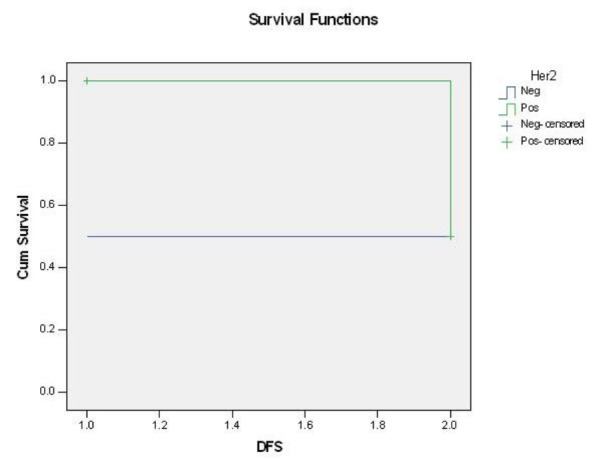
HER2/neu overexpression and disease-free survival

**Fig. 4:**
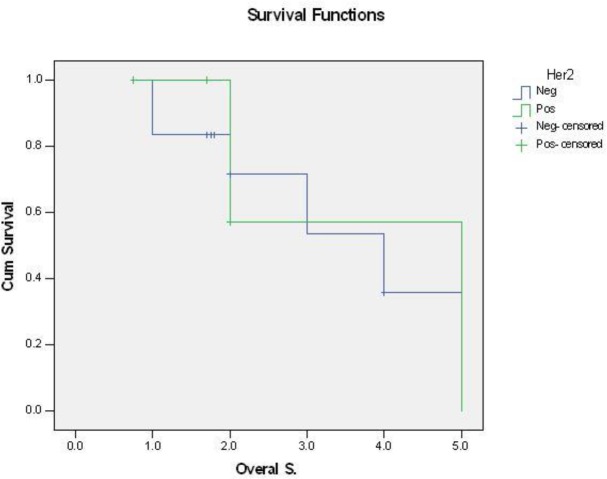
HER2/neu overexpression and overall survival

No significant correlation was recognized between the depth of myometrial invasion, vascular, perineural, and parametrial invasion and HER2/neu overexpression was seen (Tables 3 to 6). No significant correlation between the DFS and OS and HER2/neu overexpression (Figures 3 and 4).

**Table 1:** Topographic characteristics of patients and tumors

Cases (%) Characteristics	Topographic Data	Percent
Tumor Type	Papillary Serous Carcinoma	23(62.2%)
	Clear Cell Carcinoma	9(24.3%)
	Malignant Mixed Mullerian Tumor	5(24.3%)
Depth of Myometrial Invasion	≤50%	15(40.5%)
	≥50%	22(59.5%)
Cervical Stromal Involvement	Presence	11(29.7%)
Vascular Invasion	Presence	23(6202%)
Perineural Invasion	Presence	3(8.1%)
Parametrial Involvement	Presence	8(21.6%)
Presence of Endometrial Polyp	Presence	7(18.1%)
Adnexal Involvement	Presence	5(13.5%)
Omental Involvement	Presence	3(8.1%)
Pelvic Lymphnode Involvement	Presence	5(13.5%)
Tumor Stage	IA	8(21.6%)
	IB	4(10.8%)
	II	6(16.2%)
	IIIA	3(8.1%)
	IIIB	4(10.8%)
	IIIC1	7(18.9%)
	IIIC2	2(5.4%)
	IVB	3(8.1%)
Her2/neu Status	Positive	12(32.4%)

**Table 2:** Her2/neu positive according to histological type and stage

Histological Type of Tumor	Her2 Positivity	Number (%)
Papillary Serous Carcinoma	Positive	8/23(34.8%)
Clear Cell Carcinoma	Positive	2/9(22.2%)
Malignant Mixed Mullerian Tumor	Positive	2/5(40%)
Stage:		
IA	Positive	3/8(37.5%)
IB	Positive	1/4(25%)
II	Positive	2/6(33.3%)
IIIA	Positive	2/3(66.7%)
IIIB	Positive	1/4(25%)
IIIC1	Positive	1/7(14.3%)
IIIC2	Positive	1/2(50%)
IVB	Positive	1/3(33.3%)

**Table 3:** Her2/neu overexpression and depth of myometrial invasion

Type			Her2		Total
			Pos.	Neg.	
Carcinosarcoma	Depth	>50%	2 40.0%	3 60.0%	5 100.0%
	Total		2 40.0%	3 60.0%	5 100.0%
Papillary	Depth	<50%	4 36.4%	7 63.6%	11 100.0%
		>50%	4 33.3%	8 66.7%	12 100.0%
	Total		8 34.8%	15 65.2%	23 100.0
Clear cell	Depth	<50%	0 0%	4 100.0%	4 100.0%
		>50%	2 40.0%	3 60.0%	5 100.0%
	Total		2 22.2%	7 77/8%	9 100.0

**Table 4:** Her2/neu overexpression and vascular invasion

Type			Her2		Total
			Pos.	Neg.	
Carcinosarcoma	Vascular Invasion	Pos.	2 50.0%	2 50.0%	4 100.0%
		Neg.	0 0%	1 100.0%	1 100.0%
	Total		2 40.0%	3 60.0%	5 100.0%
Papillary	Vascular Invasion	Pos.	6 46.2%	7 53.8%	13 100.0%
		Neg.	2 20.0%	8 80.0%	10 100.0%
	Total		8 34.8%	15 65.2%	23 100.0
Clear Cell	Vascular Invasion	Pos.	1 16.7%	5 83.3%	6 100.0%
		Neg.	1 33.3%	2 66.7%	3 100.0%
	Total		2 22.2%	7 77/8%	9 100.0

**Table 5:** Her2/neu overexpression and perineural invasion

Type			Her2		Total
			Pos.	Neg.	
Carcinosarcoma	Perineural Invasion	Pos.	1 50.0%	1 50.0%	2 100.0%
		Neg.	1 33.3%	2 66.7%	3 100.0%
	Total		2 40.0%	3 60.0%	5 100.0%
Papillary	Perineural Invasion	Neg.	8 34.8%	15 65.2%	23 100.0
	Total		8 34.8%	15 65.2%	23 100.0
Clear Cell	Perineural Invasion	Pos.	0 0 %	1 100.0%	1 100.0%
		Neg.	1 25.0%	2 75.0%	8 100.0%
	Total		2 22.2%	7 77/8%	9 100.0

**Table 6:** Her2/neu overexpression and parametrial invasion

Type			Her2		Total
			Pos.	Neg.	
Carcinosarcoma	Parametrial Invasion	Pos.	1 50.0%	1 50.0%	2 100.0%
		Neg.	0 33.3%	2 66.7%	3 100.0%
	Total		2 40.0%	3 60.0%	5 100.0%
Papillary	Parametrial Invasion	Pos.	2 50.0%	2 50.0%	4 100.0%
		Neg.	6 31.6%	13 68.4%	19 100.0%
	Total		8 34.8%	15 65.2%	23 100.0
Clear Cell	Parametrial Invasion	Pos.	0 0%	2 100.0%	2 100.0%
		Neg.	2 28.6%	5 71.4%	7 100.0%
	Total		2 22.2%	7 77/8%	9 100.0

## Discussion

Broadly speaking, EC is a genetically heterogeneous malignancy. The dualistic model of EC, proposed by Bokhman, has been widely accepted. Based on this model, ECs are categorized into type I and type II. Type I EC includes nearly 80% of new cases and is mainly associated with a good prognosis. But type II ECs are high-grade tumors, which account for about 40% of all EC-related mortality due to aggressive biologic behavior, metastasis at the time of diagnosis, and resistance to chemotherapy (14). A contributing factor for poor prognosis of type II EC is HER2/neu overexpression, which is related to treatment resistance (11). Many studies have showed that the HER2/neu gene is attributed to the higher rate of metastasis as well as invasiveness in breast tumors (15). The role and prognostic value of HER2/neu in EC, especially in type II, has been investigated in several studies and provides knowledge for target chemotherapy. The highest HER2/neu overexpression has been reported by Santin* et al.*, in which 80% of uterine PSC showed overexpression of HER2/neu (10). In our study, the HER2/neu overexpression by IHC (2+ or 3+) and CISH was seen in 12 (32.4%) high-grade EC cases. 

Her-2/neu overexpression (2+ or 3+) rate with lowest frequency (14%) was reported by Togami *et al.* This study was the second largest study with 71 PSC tumors (1). In another study by Slomovitz* et al.*, in a series of 68 PSC cases, HER2/neu overexpression (2+ or 3+) was detected in 18% of the PSC cases with mixed or pure histologic subtypes (16). In a research by the Gynecologic Oncology Group with 38 advanced stage or recurrent PSC, HER2/neu overexpression (2+ or 3+) frequency was 61% (17). Different prevalence of HER-2/neu overexpression in previous studies can be explained by different clinical and histomorphological characteristics like the proportion of high-grade or recurrent tumors, variation in scoring and staining methods, and presence of mixed histologic subtypes in the cases of each study. The criteria for HER-2/neu expression scoring has not been defined in some studies. However, in most studies, HER-2/neu positivity criteria was complete membranous staining in more than 10% of neoplastic cells. There are variations in scoring of the cases with partial or incomplete staining of cell membranes. Inter-observer variability is another reason of difference in previous studies (10).

In the present study, HER-2/neu overexpression was seen in 34.8% of PSCs, 22.2% of CCCs, and 40% of carcinosarcomas. A wide range of HER2/neu overexpression in PSCs and CCCs has been reported in some previous studies, with ranges of 14-80% and 22-66%, respectively (8, 10).

In a study by Xiao* et al.*, HER-2/neu overexpression had significant association with higher clinical stage (III-IV), lymph node metastasis, and tumors at the G23 phase, but there was no association with age of the patient and depth of invasion (15). Another study by Morrison *et al.* showed that higher HER-2/neu expression is correlated with shorter disease-specific survival and progression-free survival in endometrial cancers; in this study, high-grade tumors and serous ECs had significantly higher HER-2/neu expression (18).

In our study, HER2/neu overexpression was increased in high-grade EC (67% in stage IIIA and 50% in stage IIIC2); however, the association between HER2/neu overexpression and tumor stage was not statistically significant. This may partly be due to the limited sample size in this study. 

Myometrial involvement was another criterion assessed in the current study. There was no statistically significant association between HER-2/neu overexpression and involvement of myometrium (more than 50%). However, we found a high rate of myometrial invasion in CCC (5 out of 9 cases showed more than 50% myometrial involvement) and 2 out of the 5 mentioned cases showed positivity for HER2/neu overexpression. 

There was also no statistically significant association between HER2/neu overexpression and vascular invasion in EC; however, vascular invasion was identified in 13 out of 23 EC cases and 6 cases showed high HER2/neu overexpression.

There was also no statistically significant correlation between perineurial invasion and HER2/neu overexpression in the current study.

In addition, concurrent presence of endometrial polyp was found in 7 out of 37 EC cases, and 3 (43%) of these tumors showed overexpressed HER2/neu.

 In present study, DFS and OS were 1.8 and 2 years, respectively. But no statistically significant correlation between DFS and HER2/neu overexpression as well as OS and HER2/neu overexpression were observed. 

With the aid of molecular studies, knowledge of the pathogenesis of EC has extensively extended over the last decade. Further stratification of EC subtypes according to their genetic alterations may improve prognostic impact and provide us with new targets for treatment. Additionally, due to unfavorable outcome in high-grade and recurrent ECs, determining biomarkers associated with better treatment response and further selection of the most effective targeted therapy have prominent clinical utility.

According to the results, it may be concluded that tumors with high HER2/neu expression constitute about one-third of high-grade ECs and they may affect the prognosis. However, further studies with larger sample size and multi-center sampling are required to achieve more accurate results. Also, determination of other contributing factors to prognosis is necessary for better programming to reduce the burden of the disease.
